# Worldwide Enucleation Techniques and Materials for Treatment of Retinoblastoma: An International Survey

**DOI:** 10.1371/journal.pone.0121292

**Published:** 2015-03-13

**Authors:** Daphne L. Mourits, Dyonne T. Hartong, Machteld I. Bosscha, Roel J. H. M. Kloos, Annette C. Moll

**Affiliations:** 1 Department of Ophthalmology, VU University Medical Center, Amsterdam, the Netherlands; 2 Department of Ophthalmology, Academic Medical Center, Amsterdam, the Netherlands; Bascom Palmer Eye Institute, University of Miami School of Medicine, UNITED STATES

## Abstract

**Purpose:**

To investigate the current practice of enucleation with or without orbital implant for retinoblastoma in countries across the world.

**Methods:**

A digital survey identifying operation techniques and material used for orbital implants after enucleation in patients with retinoblastoma.

**Results:**

We received a response of 58 surgeons in 32 different countries. A primary artificial implant is routinely inserted by 42 (72.4%) surgeons. Ten (17.2%) surgeons leave the socket empty, three (5.2%) decide per case. Other surgeons insert a dermis fat graft as a standard primary implant (n=1), or fill the socket in a standard secondary procedure (n=2; one uses dermis fat grafts and one artificial implants). The choice for porous implants was more frequent than for non-porous implants: 27 (58.7%) and 15 (32.6%), respectively. Both porous and non-porous implant types are used by 4 (8.7%) surgeons. Twenty-five surgeons (54.3%) insert bare implants, 11 (23.9%) use separate wrappings, eight (17.4%) use implants with prefab wrapping and two insert implants with and without wrapping depending on type of implant. Attachment of the muscles to the wrapping or implant (at various locations) is done by 31 (53.4%) surgeons. Eleven (19.0%) use a myoconjunctival technique, nine (15.5%) suture the muscles to each other and seven (12.1%) do not reattach the muscles. Measures to improve volume are implant exchange at an older age (n=4), the use of Restylane SQ (n=1) and osmotic expanders (n=1). Pegging is done by two surgeons.

**Conclusion:**

No (worldwide) consensus exists about the use of material and techniques for enucleation for the treatment of retinoblastoma. Considerations for the use of different techniques are discussed.

## Introduction

Retinoblastoma is a malignant intra-ocular tumor arising from the developing retina in children, mostly between the ages of 0 to 5. Treatment is primarily focused on saving the child’s life. Different treatment modalities have been developed and some offer the possibility of eye salvage [1]. Yet, enucleation, the complete removal of the eyeball, remains an important treatment modality. It is a safe method regarding survival and feasible in every hospital setting throughout the world.

Enucleation may be the oldest surgical procedure in ophthalmology [2]. Although the basics are still the same, adjustments have been made to reduce postoperative complications and improve cosmetic results.

In enucleation surgery where the socket was left empty, many patients encountered the post enucleation socket syndrome (PESS): enophthalmos, superior sulcus depression, upper- and lower lid ptosis [3, 4], and experienced little to no movement of the prosthetic eye [5]. In 1884 implants were introduced by Mules [6] for replacement of lost orbital volume, and since then changing implant shapes and materials have been used. An orbital implant can be categorized as autologous or alloplastic, integrated, semi integrated or not integrated [7]. The shape of orbital implants are usually spherical, but they can also be mounded or egg-shaped [7]. Sizes may range from 12 to 24 mm in diameter. An orbital implant may be wrapped for protection of the overlaying conjunctiva and for attachment of the rectus muscles. Wrapping materials are either organic (autologous and heterologous) or alloplastic. The rectus (and sometimes oblique) muscles may be attached to the wrapping at different positions, directly to the implant, to the fornices (myoconjunctival technique) [8], or not reattached at all. The implant can be inserted directly after enucleation (primary implant), or at a later stage (secondary implant). Nevertheless, surgery techniques and materials used for enucleation in retinoblastoma vary among surgeons and clinics. Major post-operative complications consist of socket infection, implant exposure or extrusion and socket contraction, all with a high chance of inevitable secondary surgeries. Redo surgeries in these complicated sockets are however more prone to result in a less favorable cosmetic outcome. Other complications or negative outcomes are the occurrence of the post enucleation socket syndrome, cyst formation, pyogenic granuloma, fat-graft proliferation, a smelly socket or minor prosthetic motility. Enucleation surgery in retinoblastoma patients have an increased complication risk due to adjuvant radio- or chemotherapy in a subset of patients [9]. Furthermore, enucleation at young age may lead to potential volume deficiency problems later in life [10–14]. Hence, it is important to primarily select implant materials and surgery techniques that are associated with a low chance of complications and best cosmetic outcome. Available reports suggest a difference in post-operative complications depending on the implant type and wrapping material [7]. Yet, as far as we know, no randomized controlled trials comparing different implant types and wrapping materials are available. In fact it is not known which techniques and materials are used in current practices worldwide. Awareness of available materials and specific techniques for enucleation among surgeons of different countries may result in reconsideration of local practice and cooperation to achieve worldwide consensus about the best techniques and materials.

The aim of this study is to create awareness by matters of an inventory of the currently used implant materials and surgical techniques for enucleation in retinoblastoma patients and the surgeon’s rationale for these choices.

## Methods

We designed a digital survey, mainly with multiple choice questions. Question logics were applied to filter the target group and to prevent irrelevant questions. Recipients were chosen from the author’s professional network; participants of retinoblastoma conferences (conference abstract books); corresponding authors of relevant articles and internet searches for ophthalmology, oncology and retinoblastoma websites. The survey was distributed through the online questionnaire tool ‘Surveymonkey’. Only recipients who treat retinoblastoma patients were invited to fill out the survey. All non-responders received a reminder after 1.5 months. The study was approved by the Medical Ethical Committee of the VU University Medical Center.

## Results

**Fig 1:**
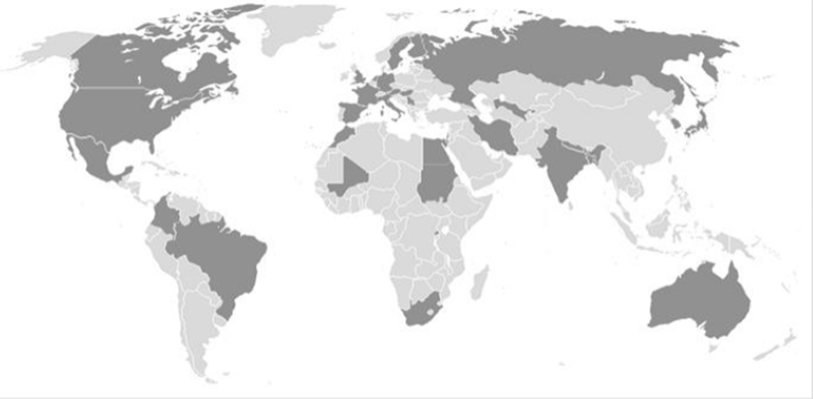
World map with included countries in dark.

The survey was initially sent to 220 recipients. Thirty invitations were bounced because of an invalid email address, 2 were rejected. Thirty-one ophthalmologists were suggested to us by other recipients. In total 68 ophthalmologists from 37 different countries responded. Five did not meet the inclusion criteria (either no treating physician of retinoblastoma or a treating physician but not performing enucleations) and five others did not complete the survey. A total of 58 surgeons of 32 different countries returned a completed survey, of whom 33 surgeons were practicing in developed countries and 25 surgeons in undeveloped or developing countries (according to the UN list and CIA (2008). "Appendix B. International Organizations and Groups." World Fact book. Retrieved 2008–04–10). See Fig. 1.

The responding surgeons diagnosed between 1 to 120 new retinoblastoma patients in the past 12 months. The mean percentage of patients who were treated by enucleation was 47.0% with a median of 47.5% (range 0–100). For developed countries the mean enucleation percentage was somewhat lower (40.9%) in comparison to the undeveloped countries (55.0%). The mean number of enucleations performed by the responding surgeon in the last 12 months was 10.2 (range 0–60 SD 11.5). Scissors were used for optic nerve dissection in 87.9% of the surgeons (n = 51), six use an enucleation snare, one uses both.

A primary artificial implant is inserted in all cases by 42 surgeons (72.4%); one inserts a primary dermis fat graft. Three surgeons (5.2%) decide whether to insert a primary implant per individual case, depending on the extra-ocular extension of the tumor. Two surgeons insert a standard secondary implant (one uses dermis fat graft, the other an artificial implant). Ten surgeons leave the socket empty.

Most surgeons who insert an artificial implant (36 of 46) use the largest implant that fits into the socket. The remaining surgeons use standard implant sizes, while some relate the size to the age of the patient (16–18–20 or 22 mm).

Porous implants are inserted by 58.7% (27 of 46) of the surgeons using artificial implants, non-porous by 32.6% (15 of 46). Both porous and non-porous implants are used by 4 surgeons. A Chi-square test for independence indicated a significant association between development of the country and choice of implant insertion X^2^ (1, *n* = 52) = 16.2, df 2, p< 0.001. Porous implants are more frequently used in developed countries. Non-porous or no implant insertion occurred more often in undeveloped countries.

Porous used implants are Polyethylene (Medpor, Porex Surgical, Newnan, Georgia, USA) (n = 11), Hydroxyapatite (Bio-eye Orbital Implants, Inc., San Diego, CA) (n = 8), Synthetic Hydroxyapatite (FCI3, Issy-Les-Mouline- aux, Cedex, France) (n = 4), Medpor SST (n = 5), Bioceramic implant (AL2O3) (n = 3), Medpor Quad Motility (n = 1) and Medpor SST Multi Purpose Conical (SST MCOI) (n = 2), Medpor Conical (COI)(n = 1).

Medpor is inserted as a bare implant by 7 out of 11 surgeons using this particular implant. Muscles are attached directly to the implant (n = 3) or to each other over the implant (n = 4). Medpor is wrapped in sclera by two other surgeons who attach the muscles to the wrapping.

Hydroxyapatite with prefab wrapping is used by five surgeons (4 polyglactin 910 mesh (Vicryl mesh; Ethicon Inc., Somerville, NJ), 1 polytetrafluoroethylene (Gore-Tex; W.L. Gore & Associates, Flagstaff, AZ), muscles are sutured to the wrapping or to the implant (at predrilled holes). One surgeon inserts bare hydroxyapatite implants and sutures the muscles to each other. Two surgeons wrap the Hydroxyapatite implant in donor sclera at which the muscles are attached. The synthetic variant (FCI3) is used by four surgeons, three with Vicryl prefab wrapping and one with donor sclera.

Three use the Bioceramic implant with prefab Vicryl wrap (n = 2) or donor sclera (n = 1).

The Medpor SST implant is used by four surgeons as a bare implant with direct muscle attachment to the implant. One wraps the Medpor SST in donor sclera and attaches the muscles to the sclera. ‘Medpor SST multipurpose’ is used by 2 surgeons (both not wrapped). The bare Medpor Quad Motility (MQM) is used by one surgeon; one wraps the Medpor conical in autologous dermis fat.

The non-porous acrylic implants are used by 11 surgeons. Eight use a bare acrylic ball, and three surgeons wrap the acrylic implants in donor sclera. One surgeon indicated to have abandoned the wrap since he changed his procedure to the myoconjunctival technique where the scleral wrap was no longer required.

Seven surgeons insert silicone implants. One wraps the silicone implant in autologous dermis fat because of the possible growth and expansion, the others use bare silicone implants.

Three surgeons insert glass balls, two as a bare implant; one wraps the glass in Gore-Tex.

Reasons for the choice of specific implant types (max. 2 reasons per surgeon) are ‘outcome’ (n = 27), this reason is most often indicated by surgeons using Medpor SST; ‘availability’ (n = 18) most for acrylic implants; ‘costs’ (n = 16) also most acrylic implants; ‘experience’ (n = 14); ‘ease of use’ (n = 11 of which 10 mentioned by Medpor (normal, SST and MCOI) users and ‘protocol’ (n = 1).

Bare implants are inserted by 25 surgeons (54.3%). Wrappings are used by 21 surgeons (of which 8 addressed to prefab wrappings, 10 to scleral wraps, 1 separate Vicryl mesh, 1 separate Gore-Tex and 1 dermis fat graft wrap). Reasons for the wrappings are ‘the implant was already wrapped in this material’ (n = 6), ‘ease of use’ (n = 3, all prefab), ‘outcome’ (n = 4), ‘experience’ (n = 2), and ‘costs’ (n = 4). The latter referred to donor scleral wrapping.

During implant insertion the muscles are attached to the wrapping (n = 13); attached directly to the implant (n = 12); attached to the fornices with the myoconjunctival technique (n = 7); sutured to each other (n = 6), either implant or wrapping depending on implant type (n = 4) or not reattached (n = 4). The location of attachment to the wrapping and/ or implant is paracentral (n = 17) or corners to each other (n = 6) and central over each other (n = 1). One surgeon sutures the muscles to the wrapping with the corners attached to each other, but places the superior rectus muscle more posteriorly to avoid ptosis. Another surgeon uses the same technique but attaches the 3 other muscles at the original muscle insertion.

All different combinations of implants, wrappings and muscle attachment are shown in Table 1.

**Table 1:** Combinations of implants, wrappings and muscle attachment.

Implant type	Implant	Wrapping	Muscle attachment	N.	Countries
**Non-porous**	Glass	Gore-tex	H	1	undeveloped
		No wrapping	E	1	undeveloped
			E	1	developed
	Acrylic	Donor sclera	A	2	developed
			C	1	undeveloped
		No wrapping	A	1	undeveloped
			B	1	undeveloped
			E	1	developed
			E	3	undeveloped
			F	1	undeveloped
			H	1	undeveloped
	Silicone	Autologous dermis fat	C	1	developed
		No wrapping	G	1	undeveloped
			E	2	undeveloped
			F	1	undeveloped
			F	2	developed
**Porous**	HA	Donor sclera	B	1	undeveloped
			D	1	developed
		Gore-tex	C	1	developed
		Vicryl	C	4	developed
		No wrapping	B	1	undeveloped
	FCI3	Donor sclera	A	1	developed
		Vicryl	G	1	undeveloped
			A	1	developed
			C	1	developed
	Bioceramic	Donor sclera	C	1	developed
		Vicryl	C	2	developed
	Medpor	Donor sclera	C	1	developed
			C	1	undeveloped
		Vicryl	C	1	developed
			D	1	undeveloped
		No wrapping	A	2	developed
			B	2	undeveloped
			B	2	developed
			C	1	undeveloped
	Medpor SST	Donor sclera	C	1	developed
		No wrapping	A	1	developed
			C	3	developed
	MQM	No wrapping	B	1	developed
	MCOI	Autologous dermis fat	D	1	undeveloped
	SST MCOI	No wrapping	B	1	developed
			C	1	developed
**Other**	No implant	No wrapping	B	2	developed
			B	2	undeveloped
			E	3	undeveloped
			F	3	undeveloped
	Dermis fat graft	No wrapping	C	1	developed
			E	1	undeveloped

***N** = number of surgeons using this method and material. (Total number is >58 because some surgeons use more than one material/ method). **Muscle attachment***: **A** Corners attached to each other; **B** Muscles overlapping/ sutured over each other; **C** Paracentral; **D** Original muscle insertion; **E** Myoconjunctival technique; **F** Loose; **G** like A (corners attached to each other), but rectus superior more posteriorly; **H** like D (original muscle insertion), but rectus superior more posteriorly (for images of different muscle locations, see S1 Fig). ***Implants***: **Medpor SST** = Medpor smooth surface tunnel; **MCOI** = Medpor conical; **MQM** = Medpor quad motility; **SST MCOI** = smooth surface tunnel medpor multipurpose conical; **HA** = Hydroxyapatite (Bio-eye); **FCI3** = Synthetic Hydroxyapatite.

The reason for a standard dermis fat graft was ‘outcome’ for both surgeons using the autologous implants. One also mentions ‘the costs’ and the other reports ‘good experience’ with dermis fat grafts. Muscles are attached to the dermis fat graft (n = 1) or attached to the fornices (n = 1), also referred to as myoconjunctival technique.

Ten surgeons never insert an implant of whom eight practice in under developed countries. Main reason (n = 8) is ‘no implant insertion for follow up regarding tumor recurrence‘, other reasons are: ‘costs’ (n = 1), ‘lack of availability’ (n = 1), ‘lack of difference in cosmetic outcome’ (n = 1), ‘lacking experience’ (n = 1), ‘no formally approved implant in this country’ (n = 1) and ‘radiotherapy after surgery’ (n = 1). The rectus muscles are not reattached (n = 3), sutured to the fornixes (n = 3) or sutured to each other as a knot (n = 4).

Additional measures for orbital growth stimulation are taken by 9 surgeons. Four replace the implant at older age, one often inserts dermis fat grafts, one did not specify and one mentions the repetitive refitting of the ocular prosthesis. Injectables ‘Restylane SQ’ and osmotic tissue expanders are used by two different surgeons, both in combination with orbital implants (Medpor SST and Medpor).

Two surgeons peg the orbital implant (one pegs a solid silicone implant, one a porous Medpor SST).

## Discussion

Our results demonstrate a wide difference in the used implant types and enucleation techniques in retinoblastoma patients. Every surgeon has his reasons for particular materials and techniques: costs, availability, experience, recurrence risk, cosmetic outcome. It seems that in most hospitals a certain protocol is followed based on agreement of surgeons working in the same hospital. However, different techniques are sometimes seen between surgeons within the same hospital. Only one surgeon mentioned ‘a protocol’ as reason for the specific implant choice. It is obvious that an international protocol does not exist, let alone consensus on a golden standard.

Most surgeons who do not insert an implant practice in undeveloped countries (8 of 10). This might be explained by their socio-economic status. In the past we did not insert orbital implants in patients enucleated for the treatment of a tumor, because the implant would interfere with palpation of the socket and clinical detection of recurrence. Today with MRI techniques (which enables detection of tumor recurrence with an orbital implant in situ) and better understanding of histopathological risk factors for tumor recurrence, implant insertion does not need to be avoided anymore [15]. Implant insertion does however bring more costs: first the implant itself and second the possible need for MRI.

‘Follow up’ was mentioned as reason to leave the socket empty by seven out of eight surgeons of the undeveloped countries, but also by one surgeon in the developed countries. One surgeon does not insert an implant when radiotherapy is required. Although radiotherapy increases socket complication rates such as socket contraction and orbital implant insertion is known to prevent socket contraction [9, 16]. We experienced this positive effect of an orbital implant with respect to socket complications in our subpopulation of radiated retinoblastoma cases.

Surgeons from developed countries most often use porous implants. The porous implants continue to be altered with (theoretical) improvements, but subsequently higher costs. Therefore, innovation and experimenting with new implants is more feasible for the developed countries.

No randomized controlled trials and very few controlled studies [17–19] have been done in this population to compare non-porous implants with the newer porous implants [20]. So far, no proven superiority for the new generation implants is demonstrated [21–23]. The motility of artificial eyes in patients with unpegged porous implants have been proven similar to that in patients with non porous implants [18, 19].

Pegging is rarely performed by our respondents. This is conform results of general enucleation/evisceration surveys [17, 24]. The previously suggested advantage of pegging (improved motility) was outbalanced by the experienced disadvantages [25] (high complication rates, especially infections and exposure).

Outcome as reason to use a specific implant was most frequently mentioned in relation to Medpor SST. Results of Medpor SST after enucleation for retinoblastoma is as far as we know only published by Choi et al. [26] They do indeed report a good outcome with the Medpor SST with no cases of implant exposure, extrusion or infection in a population of 44 children treated for unilateral retinoblastoma, with 34.1% additionally treated with chemotherapy and none with radiotherapy [26].

Availability and costs were the main arguments for choice of implant by solid implant users. The argument of availability was given by 9 of 20 (45.0%) surgeons of developed countries inserting implants and 9 of 26 (34.6%) of undeveloped countries. Some surgeons using the more sophisticated implants also mentioned ‘availability’. Costs were mentioned by 12 of 26 (46.2%) undeveloped countries and 4 of 20 (20%) developed countries.

Regarding the ease of use of the different implants the Medpor implants (including subtypes) seem to be most convenient. As for the Medpor SST this can be subscribed to the features of the predrilled tunnels for the muscles and the smooth anterior surface enabling insertion without wrapping. The reason for the frequently argued easy use of the normal Medpor (without the tunnels and with need for wrapping) is not clear to us.

Remarkable was the response of one practice from a developed country who inserts bare silicone implants without attachment of the muscles reasoning the experience and outcome. The use of bare silicone implant is reported by Wells et al. [27] with good results in 73 of 75 cases (no migration, exposure or extrusion). Yet muscles were attached directly to the silicone implant. As far as we know, no literature is available on the technique using silicone implant without reattachment of the muscles.

The myoconjunctival technique is performed by a substantial number of surgeons from both developed and undeveloped countries, with different solid implants and when no implant is inserted. This technique is based on the theory that prosthesis motility is not only a result of the implant motility but also provided by contraction of the fornices. Shome et al. [28] compared in a randomized controlled trial with >1 year follow up the traditional muscle imbrication technique using polymethyl methacrylate (PMMA) (n = 50) or porous polyethylene (n = 50) and myoconjunctival technique (n = 50) using PMMA implants. Results of this study demonstrated statistically and clinically significant better implant and prosthesis movement with the PMMA myoconjunctival technique [28]. Yadava et al. [8] confirmed in a randomized controlled trial with 30 patients the superiority of the myoconjunctival technique. The location of muscle attachment plays a role in occurrence of complications. Although different locations of muscle attachment to implant (in predrilled holes or tunnels) or to the wrapping are mentioned, studies on this subject are scarce. When muscles are imbricated the implant may migrate [29]. Custer et al. [30] reported fewer exposures when suturing the muscles anterior to their standard location. The relation between position of superior muscle attachment to the implant and postoperative ptosis is not reported in the literature. The two surgeons who reported suturing the rectus superior more posteriorly may speak from their own experience, but this is an interesting concept, since the levator complex is potentially less affected with the adapted position. Yet, ptosis following enucleation is regarded as part of the post enucleation socket syndrome, when no sufficient volume is substituted [31]. The orbital contents rotatory displace from superior to posterior and from posterior to inferior. This redistribution of orbital fat appears to result from internal retraction of the superior muscle complex [4]. Kaltreider et al. [32] retrospectively studied 138 anophthalmic patients (94 with ptosis, 44 without ptosis) and reported in accordance with Vistnes [31] a direct relation between the substituted orbital volume and the occurrence of ptosis, the more volume replacement, the smaller the odds of ptosis [32]. Kaltreider et al. [32] postulated no relation was found between muscle imbrication and occurrence of ptosis, however the number of patients with imbrication counted only 6 in the ptosis group and 2 in the control group. Kim et al. [33] describe a measured decrease of levator function in anophthalmic sockets, not because of actual loss of function but because of its change in contour and decrease of length resulting in lower resting tension.

The purpose of this survey was to create awareness about the current state of the art of enucleation for retinoblastoma worldwide. The methods used by surgeons across the world are even more diverse than we expected, and we have learned interesting opinions from experienced surgeons. Yet, a comparison between techniques cannot be made because of this large diversity in techniques in a small number of patients, and a lack of prospective studies. Therefore we propose international collaboration to enable prospective studies for the comparison of selected types of enucleation techniques and implant materials.

## Supporting Information

S1 Fig Images of different muscle locations. (DOCX)

S1 Dataset Full dataset anonymized. (XLSX)

## References

[1] Shields CL, Shields JA. Retinoblastoma management: advances in enucleation, intravenous chemoreduction, and intra-arterial chemotherapy. Curr Opin Ophthalmol. 2010; 21(3): 203–12. 10.1097/ICU.0b013e328338676a 20224400

[2] Moshfeghi DM, Moshfeghi AA, Finger PT. Enucleation. Surv Ophthalmol. 2000; 44(4): 277–301. 10667436 10.1016/s0039-6257(99)00112-5

[3] Tyers AG, Collin JR. Orbital implants and post enucleation socket syndrome. Trans Ophthalmol Soc UK. 1982; 102: 90–2. 6963069

[4] Smit TJ, Koornneef L, Zonneveld FW, Groet E, Otto AJ. Computed tomography in the assessment of the postenucleation socket syndrome. Ophthalmology. 1990; 97(10): 1347–51. 2243686 10.1016/s0161-6420(90)32411-9

[5] Smit TJ, Koornneef L, Groet E, Zonneveld FW, Otto AJ. Prosthesis motility with and without intraorbital implants in the anophthalmic socket. Br J Ophthalmol. 1991; 75(11): 667–70. 1751462 10.1136/bjo.75.11.667 PMC1042525

[6] Mules PH. Evisceration of the globe, with artificial vitreous. Trans Ophthalmol Soc UK. 1885; 5: 200.

[7] Baino F, Perero S, Ferraris S, Miola M, Balagna C, Verné E, et al Biomaterials for orbital implants and ocular prostheses: overview and future prospects. Acta Biomater. 2014; 10(3): 1064–87. 10.1016/j.actbio.2013.12.014 24342039

[8] Yadava U, Sachdeva P, Arora V. Myoconjunctival enucleation for enhanced implant motility. result of a randomised prospective study. Indian J Ophthalmol. 2004; 52(3): 221–6. 15510462

[9] Shildkrot Y, Kirzhner M, Haik BG, Qaddoumi I, Rodriguez-Galindo C, Wilson MW. The effect of cancer therapies on pediatric anophthalmic sockets. Ophthalmology. 2011; 118(12): 2480–6. 10.1016/j.ophtha.2011.05.024 21856015 PMC3539308

[10] Hintschich C. Bony orbital development after early enucleation in humans. Br J Ophthalmol. 2001; 85(2): 205–8. 11159487 10.1136/bjo.85.2.205 PMC1723856

[11] Chojniak MM, Chojniak R, Testa ML, Min TT, Guimarães MD, Barbosa E, et al Abnormal orbital growth in children submitted to enucleation for retinoblastoma treatment. Pediatr Hematol Oncol. 2012; 34(3): 102–5. 10.1097/MPH.0b013e318236c346 22258347

[12] Peylan-Ramu N, Bin-Nun A, Skleir-Levy M, Bibas A, Koplewitz B, Anteby I, et al Orbital growth retardation in retinoblastoma survivors: work in progress. Med Pediatr Oncol. 2001; 37(5): 465–70. 11745876 10.1002/mpo.1231

[13] Fountain TR, Goldberger S, Murphree AL. Orbital development after enucleation in early childhood. Ophthal Plast Reconstr Surg. 1999; 15(1): 32–6. 9949427 10.1097/00002341-199901000-00008

[14] Kaltreider SA, Peake LR, Carter BT. Pediatric enucleation: analysis of volume replacement. Arch Ophthalmol. 2001; 119(3): 379–84. 11231771 10.1001/archopht.119.3.379

[15] Carroll WL, Finlay JL. Cancer in Children and Adolescents. Jones & Bartlett Publishers; 2010.

[16] Sami D, Young S, Petersen R. Perspective on orbital enucleation implants. Surv Ophthalmol. 2007; 52(3): 244–65. 17472801 10.1016/j.survophthal.2007.02.007

[17] Viswanathan P, Sagoo MS, Olver JM. UK national survey of enucleation, evisceration and orbital implant trends. Br J Ophthalmol. 2007; 91(5): 616–9. 17151061 10.1136/bjo.2006.103937 PMC1954760

[18] Colen TP, Paridaens DA, Lemij HG, Mourits MP, van Den Bosch WA. Comparison of artificial eye amplitudes with acrylic and hydroxyapatite spherical enucleation implants. Ophthalmology. 2000; 107(10): 1889–94. 11013194 10.1016/s0161-6420(00)00348-1

[19] Custer PL, Trinkaus KM, Fornoff J. Comparative motility of hydroxyapatite and alloplastic enucleation implants. Ophthalmology. 1999; 106(3): 513–6. 10080207 10.1016/S0161-6420(99)90109-4

[20] Mourits MP. A Short History of Contemporary Oculoplastic Surgery (and the Need for RCTs): Excerpts from the Mustardé Lecture 2011. Orbit. 2012; Early Online: 1–4. 10.3109/01676830.2011.638098 22616612

[21] Nunery WR, Heinz GW, Bonnin JM, Martin RT, Cepela MA. Exposure rate of hydroxyapatite spheres in the anophthalmic socket: histopathologic correlation and comparison with silicone sphere implants. Ophthal Plast Reconstr Surg. 1993; 9: 96–104. 8391837 10.1097/00002341-199306000-00004

[22] Trichopoulos N, Augsburger JJ. Enucleation with unwrapped porous and nonporous orbital implants: a 15-year experience. Ophthal Plast Reconstr Surg. 2005; 21(5): 331–6. 16234693 10.1097/01.iop.0000175034.88019.a5

[23] Cleres B, Meyer-Rüsenberg HW. Porous orbital implants. Ophthalmologe. 2014; 111(6): 572–6. 10.1007/s00347-013-2950-7 24942122

[24] Su GW, Yen MT. Current trends in managing the anophthalmic socket after primary enucleation and evisceration. Ophthal Plast Reconstr Surg. 2004; 20(4): 274–80. 15266140 10.1097/01.iop.0000129528.16938.1e

[25] Jordan DR, Chan S, Mawn L, Gilberg S, Dean T, Brownstein S, et al Complications associated with pegging hydroxyapatite orbital implants. Ophthalmology. 1999; 106(3): 505–12. 10080206 10.1016/S0161-6420(99)90108-2

[26] Choi YJ, Park C, Jin HC, Choung H-K, Lee MJ, Kim N, et al Outcome of smooth surface tunnel porous polyethylene orbital implants (Medpor SST) in children with retinoblastoma. Br J Ophthalmol. 2013; 97(12): 1530–3. 10.1136/bjophthalmol-2013-303481 24064935

[27] Wells TS, Harris GJ. Direct Fixation of Extraocular Muscles to a Silicone Sphere: A Cost-Sensitive, Low-Risk Enucleation Procedure. Ophthal Plast Reconstr Surg. 2011; 27(5): 364–7. 10.1097/IOP.0b013e31821c1298 21629135

[28] Shome D, Honavar SG, Raizada K, Raizada D. Implant and prosthesis movement after enucleation: a randomized controlled trial. Ophthalmology. 2010; 117(8): 1638–44. 10.1016/j.ophtha.2009.12.035 20417565

[29] Allen L. The argument against imbricating the rectus muscles over spherical orbital implants after enucleation. Ophthalmology. 1983; 90(9): 1116–20. 6646651 10.1016/s0161-6420(83)80055-4

[30] Custer PL, Kennedy RH, Woog JJ, Kaltreider SA, Meyer DR. Orbital implants in enucleation surgery: a report by the American Academy of Ophthalmology. Ophthalmology. 2003; 110(10): 2054–61. 14522788 10.1016/S0161-6420(03)00857-1

[31] Vistnes LM. Mechanism of upper lid ptosis in the anophthalmic orbit. Plast Reconstr Surg. 1976; 58(5): 539–45. 981399 10.1097/00006534-197611000-00002

[32] Kaltreider SA, Shields MD, Hippeard SC PJ. Anophthalmic Ptosis: Investigation of the Mechanisms and Statistical Analysis. Ophthal Plast Reconstr Surg. 2003; 19(6): 421–8. 14625487 10.1097/01.IOP.0000092799.82563.D7

[33] Kim NJ, Khwarg SI. Decrease in levator function in the anophthalmic orbit. Ophthalmologica. 2008; 222(5): 351–6. 10.1159/000146896 18645260

