# Supplementary figures and images for: Worldwide Enucleation Techniques and Materials for Treatment of Retinoblastoma: An International Survey
Source: PLoS One. 2015 Mar 13;10(3):e0121292. doi: 10.1371/journal.pone.0121292 (PMC4358947; doi:10.1371/journal.pone.0121292)

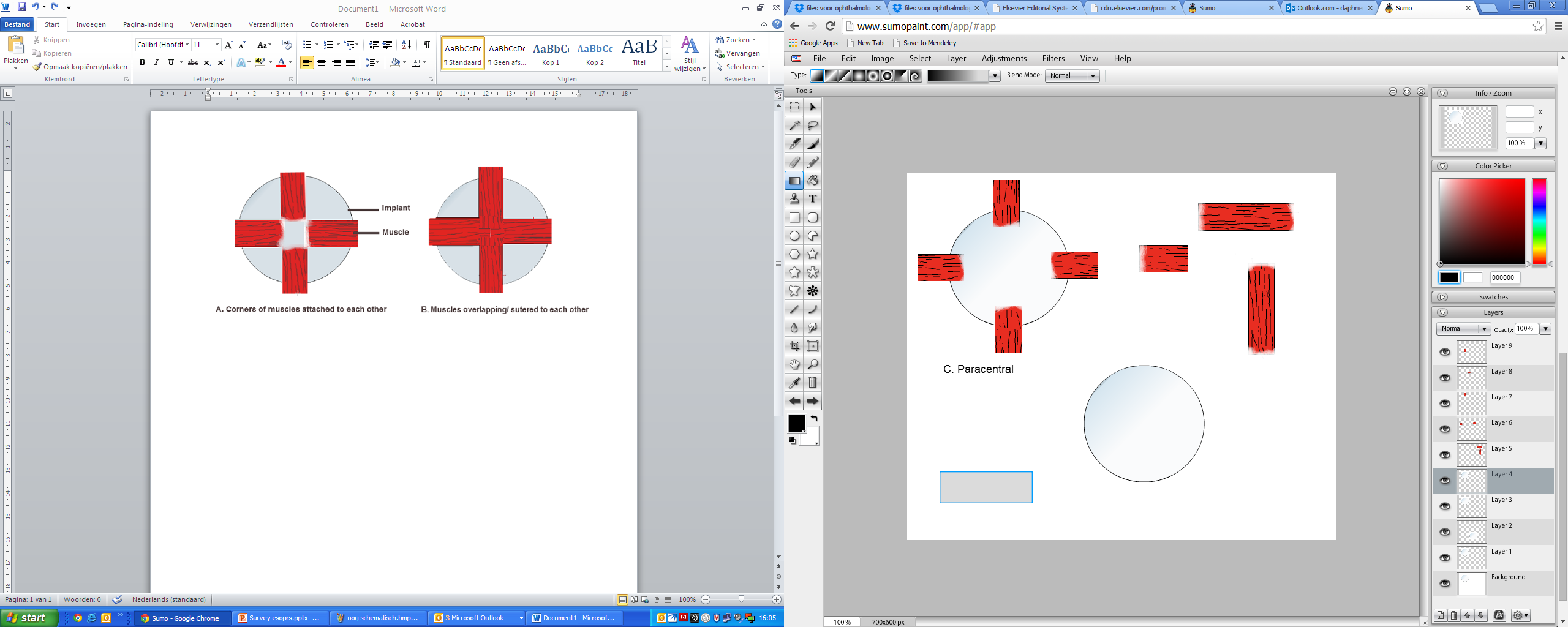

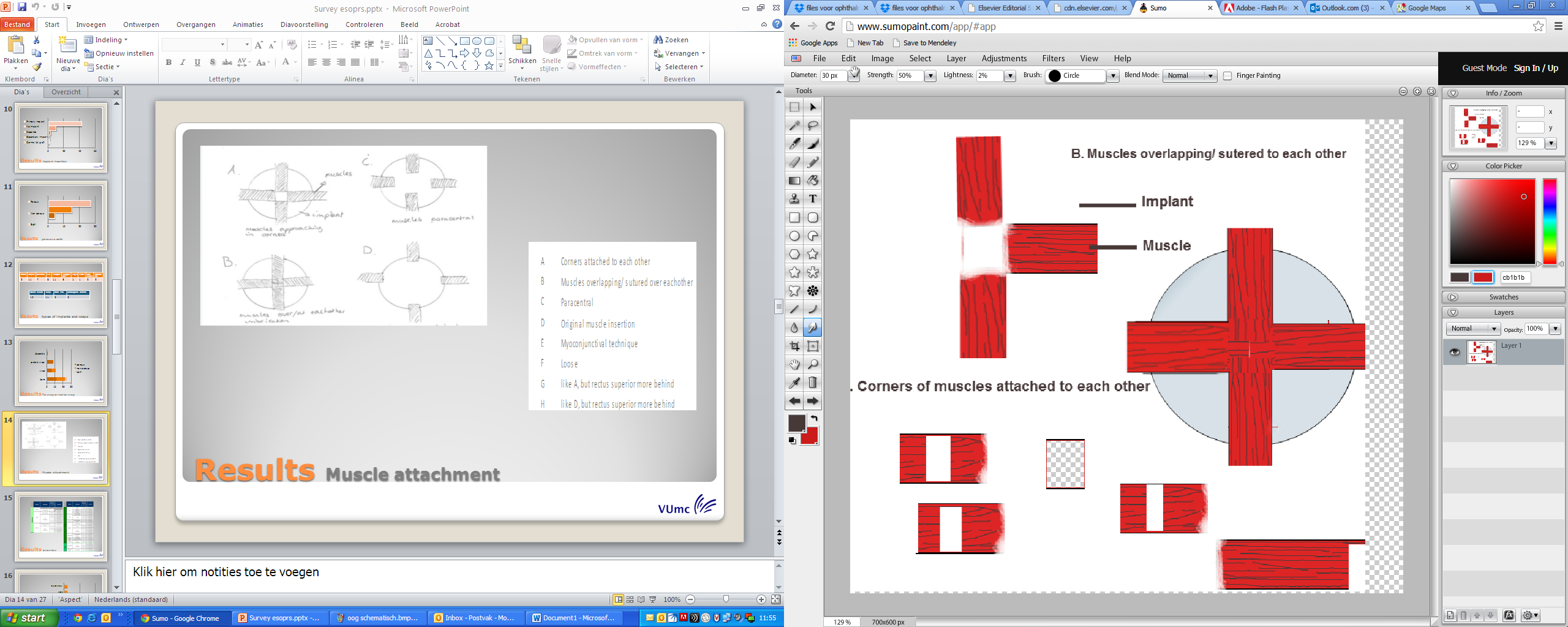

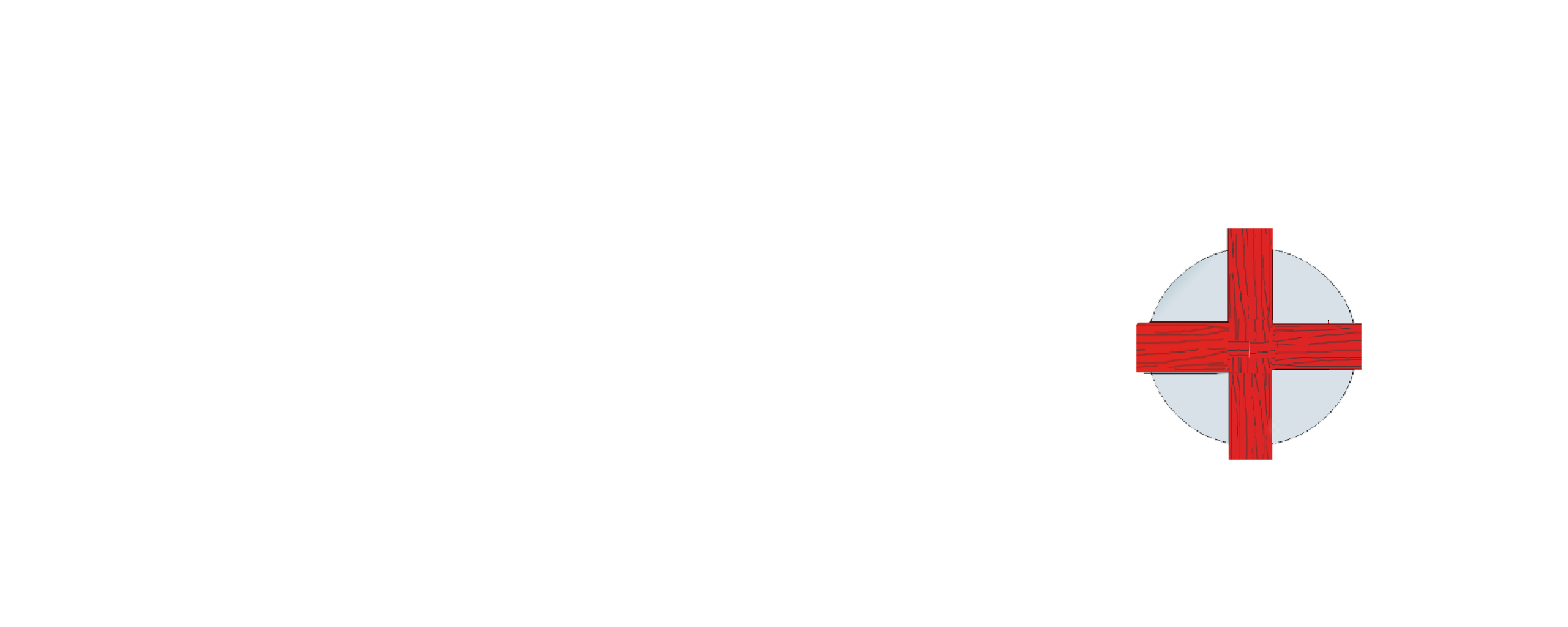

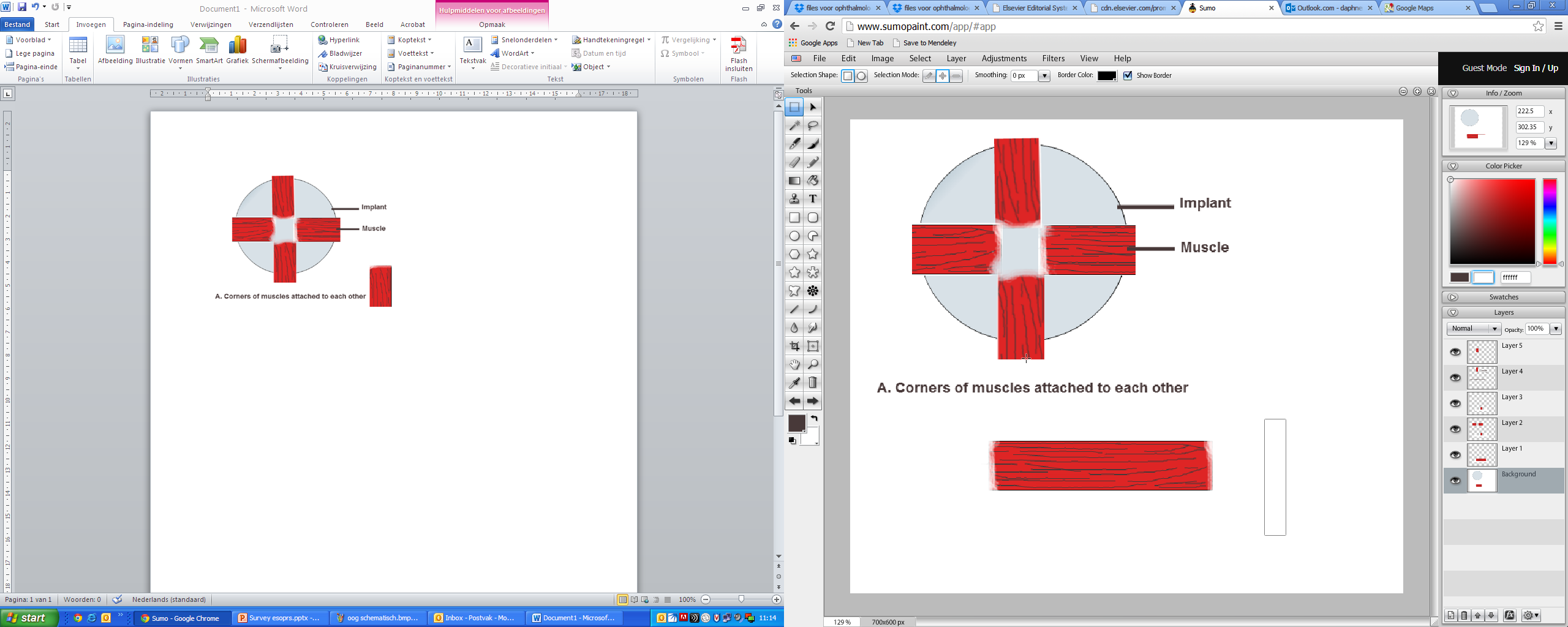


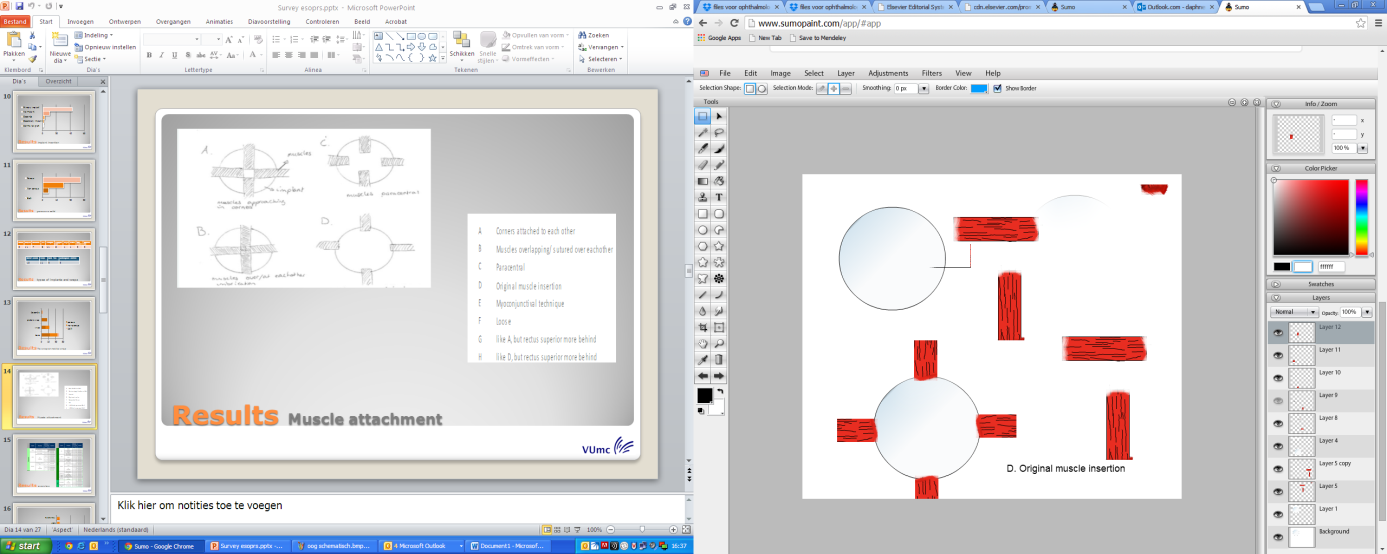


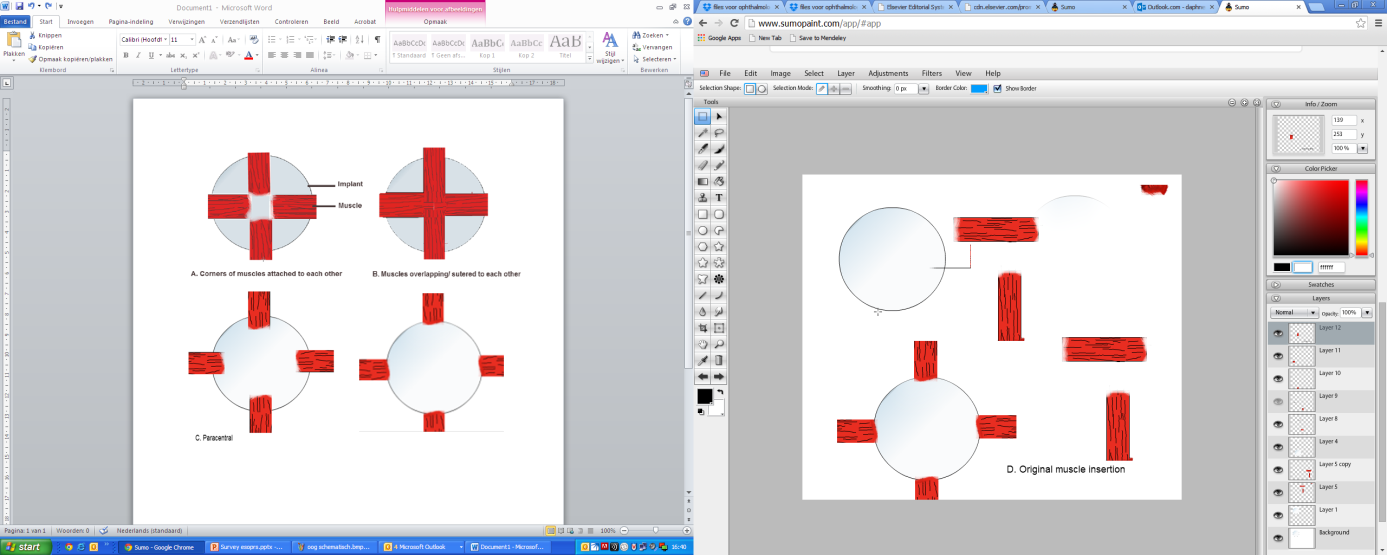

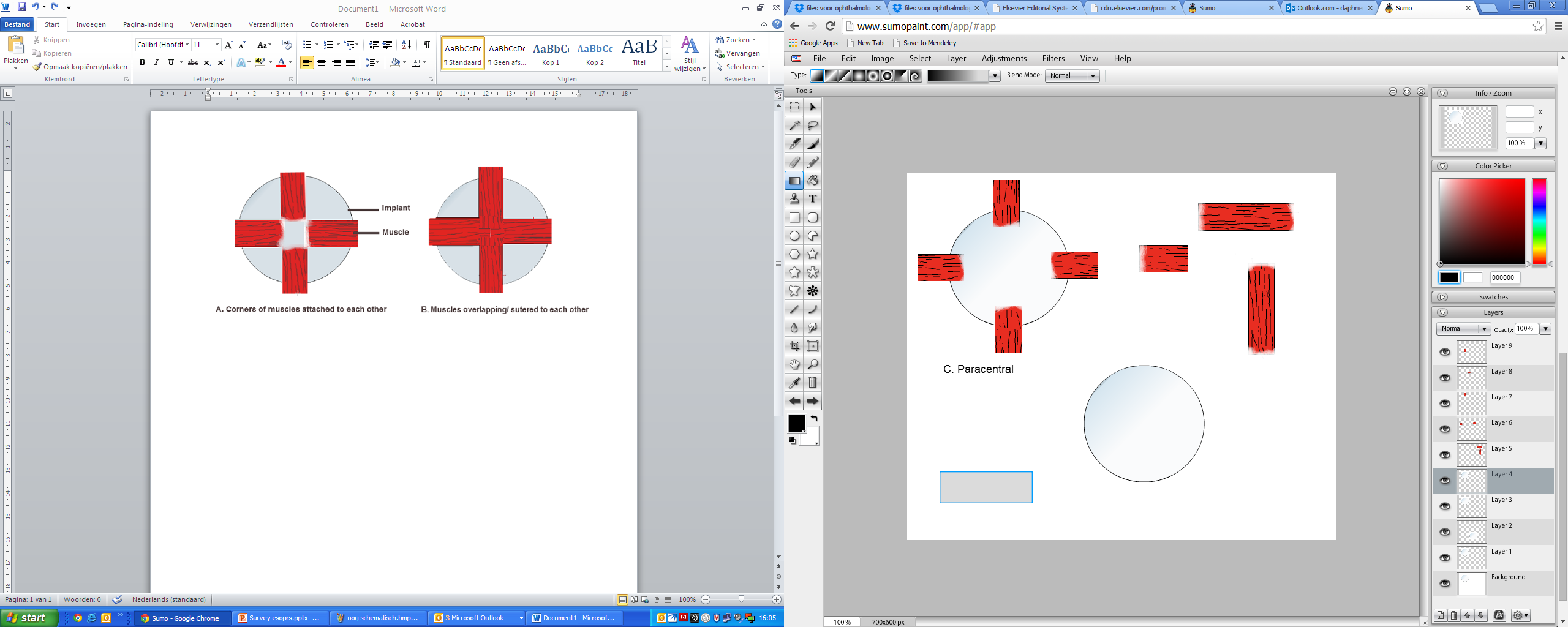

Supplement: S1 Fig — (DOCX) [file pone.0121292.s001.docx]
